# Development and Optimization of Oligonucleotide Ligation Assay (OLA) Probes for Detection of HIV-1 Resistance to Dolutegravir

**DOI:** 10.3390/v16071162

**Published:** 2024-07-19

**Authors:** Ingrid A. Beck, Ceejay L. Boyce, Marley D. Bishop, Yen L. Vu, Amanda Fung, Sheila Styrchak, Nuttada Panpradist, Barry R. Lutz, Lisa M. Frenkel

**Affiliations:** 1 Center for Global Infectious Disease Research, Seattle Children’s Research Institute, Seattle, WA 98109, USA; ingrid.beck@seattlechildrens.org (I.A.B.); ceejay.boyce@seattlechildrens.org (C.L.B.); marley.bishop@seattlechildrens.org (M.D.B.); sheila.styrchak@seattlechildrens.org (S.S.); 2 Department of Bioengineering, University of Washington, Seattle, WA 98195, USA; vuyenlan@uw.edu (Y.L.V.); afung00@uw.edu (A.F.); nuttadap@uw.edu (N.P.); blutz@uw.edu (B.R.L.); 3 Departments of Medicine, Pediatrics and Laboratory Medicine, University of Washington, Seattle, WA 98195, USA

**Keywords:** HIV-1 integrase, dolutegravir, INSTI resistance, drug resistance mutations, drug resistance testing, oligonucleotide ligation assay

## Abstract

The WHO currently recommends dolutegravir (DTG)-based ART for persons living with HIV infection in resource-limited-settings (RLS). To expand access to testing for HIV drug resistance (DR) to DTG in RLS, we developed probes for use in the oligonucleotide ligation assay (OLA)-Simple, a near-point of care HIV DR kit. Genotypic data from clinical trials and case reports were used to determine the mutations in HIV-1 integrase critical to identifying individuals with DTG-resistance at virologic failure of DTG-based ART. Probes to detect G118R, Q148H/K/R, N155H and R263K in HIV-1 subtypes A, B, C, D and CRF01_AE were designed using sequence alignments from the Los Alamos database and validated using 61 clinical samples of HIV-1 subtypes A, B, C, D, CRF01_AE genotyped by PacBio (*n* = 15) or Sanger (*n* = 46). Initial OLA probes failed to ligate for 16/244 (6.5%) codons (9 at G118R and 7 at Q148H/K/R). Probes revised to accommodate polymorphisms interfering with ligation at codons G118R and Q148R reduced indeterminates to 3.7% (5 at G118R and 4 at Q148H/K/R) and detected DTG-mutations with a sensitivity of 96.5% and 100% specificity. These OLA DTG resistance probes appear highly sensitive and specific across HIV-1 subtypes common in RLS with high burden of HIV infection.

## 1. Introduction

High rates of human immunodeficiency virus type-1 (HIV) drug resistance (DR) to non-nucleoside reverse transcriptase inhibitors (NNRTIs) led the World Health Organization (WHO) in 2019 to switch the recommended first-line antiretroviral therapy (ART) from tenofovir/lamivudine/efavirenz (TLE) to dolutegravir (DTG)-based ART [1]. DTG, a second-generation integrase strand transfer inhibitor (INSTI), is highly effective and has a relatively high barrier to HIV drug resistance [2,3]. Following this WHO recommendation and the availability of relatively low cost tenofovir/lamivudine/DTG (TLD), public health programs in resource-limited settings (RLS) rolled-out DTG-based regimens, which by 2024 have reached most persons living with HIV (PLWH) [4,5]. 

In clinical trials of DTG-based ART, low rates of virological failure were observed with rare emergence of INSTI-associated DR mutations [6,7,8,9]. However, more recent “real world” observational studies in RLS report relatively higher rates of DTG resistance [10,11,12,13,14], particularly among individuals with previous virologic failure who switched to TLD [14,15]. Factors that might facilitate the emergence and spread of DTG resistance in RLS include impediments to ART-adherence, such as drug stock-outs, stigma and other social conditions [16], and/or clinical parameters, such as high baseline plasma HIV RNA, low CD4 cell counts, active opportunistic infections, and drug interactions [17,18]. While virologic failure of prior ART is associated with increased rates of DTG resistance, the effects of resistance to specific nucleoside reverse transcriptase inhibitors (NRTI) on DTG-based ART efficacy and the selection of DTG DR vary. High rates of NRTI resistance have been observed in people failing first-line NNRTI-ART [8,11] (e.g., 92% and 60% to lamivudine; and 58% and 47% to tenofovir). However, some studies find significant associations between NRTI mutations and subsequent DTG resistance [10,14], and others do not [8,11]. The association of NRTI resistance with DTG DR is of concern in RLS due to the recycling of tenofovir and lamivudine in TLD taken as the first or second-line regimen. Additional randomized studies seem warranted, as in observational studies of individuals switching from first-line TLE to TLD without viral load or DR testing [19], a subset of individuals were unknowingly switched while experiencing virologic failure with resistance to tenofovir and/or lamivudine which may continue adherence patterns that place them at increased risk of virologic failure and development of DTG resistance [17]. 

Access of PLWH to DR monitoring is limited in most RLS due to the cost and infrastructure needed for Sanger sequencing, the gold standard for HIV DR testing. When available, DR testing is usually performed in centralized laboratories which can result in prolonged “turnaround times” of weeks to months for test results to reach clinicians and PLWH [20]. This in turn can delay needed adjustments to ART regimens and facilitate the accumulation of resistance mutations and transmission of drug resistant variants. 

Our group developed OLA-Simple, a laboratory-based point mutation oligonucleotide ligation assay (OLA), that detects HIV DR mutations in reverse transcriptase associated with failure of the previous first-line NNRTI-based ART (tenofovir, abacavir, lamivudine and efavirenz) [21,22,23,24]. OLA-Simple kits provide ready-to-use lyophilized reagents, lateral flow strips and step-by-step software; these simplify laboratory procedures, allow visual or digital interpretation of results and reduce the time and technical expertise needed to perform the assay [22]. In addition to these attributes, OLA-Simple requires relatively inexpensive equipment often found in community laboratories (thermocyclers, micropipettes, office scanner) making OLA-Simple suitable for decentralized laboratories [25]. 

OLA-Simple for NRTI and NNRTI mutations detects HIV DR across multiple HIV-1 subtypes including A, B, C, D and CRF01_AE and has shown high sensitivity and specificity compared to consensus sequencing in clinical specimens from Kenya, Peru, Thailand, South Africa, and Mexico [22,23]. With the goal of facilitating access to DR monitoring of DTG-based ART in RLS, we developed and optimized a novel set of probes to detect resistance to DTG-based ART across HIV subtypes A, B, C, D and CRF01_AE. 

## 2. Materials and Methods

### 2.1. Selection of HIV-1 Integrase Mutations to Include in the OLA-Simple Kit

We compiled HIV genotypic data from clinical trials and case reports that enrolled INSTI-naïve participants and described outcomes of DTG-based ART. The most prevalent INSTI-resistance mutations, as defined by the Stanford HIV Drug Resistance Database [26,27], reported in those failing DTG-based ART were tabulated. The major INSTI DR mutations that identified the majority of participants with DTG resistance at virologic failure were determined using participant-level mutations. These mutations were deemed “OLA DTG resistance mutations”.

### 2.2. Design of OLA Probes

Ligation probes were designed to detect OLA DTG resistance mutations in HIV-1 subtypes A, B, C, D and CRF01_AE using sequence alignments from the Los Alamos National Laboratory HIV Database (LANL, https://www.hiv.lanl.gov, accessed on 1 February 2022). To address HIV sequence diversity, probes included mixed nucleotides at sites of prevalent polymorphisms or mixtures of probes optimized for specific HIV subtypes. After testing of these probes with remanent clinical specimens, the sequences of specimens that yielded indeterminate OLA results (see below) were reviewed for polymorphic positions near the probes’ ligation site, and when detected, additional probes were designed to accommodate these polymorphisms. HPLC purified probes were obtained from Integrated DNA Technologies (Coralville, IA, USA).

### 2.3. Laboratory-Based OLA

A laboratory ELISA-based OLA was used to optimize assay conditions (i.e., probe and ligase concentrations) for each DTG targeted mutation to achieve detection of mutant frequencies of ≥2% in the viral population as previously described [28]. Mutation-specific standard curves were prepared using mixtures of wildtype and mutant plasmids containing the mutation of interest at concentrations of 0, 2, 5, 10, 25, 50, 75 and 100% mutant. Standard curves were run in each OLA assay and the optical density (OD) of the 2% mutant control was used as the mutant positive cut-off for each mutation tested. Samples with negative mutant results (OD < 2% mutant control) and wildtype results with an OD < 30% of the 100% wildtype control were considered to have indeterminate OLA results (IND). Remanent samples from individuals viremic during DTG-based ART and untreated individuals were assayed in duplicate. Mutations Q148H, Q148K and Q148R were tested in separate OLA assays.

### 2.4. Assessment of OLA Probes in Clinical Specimens

To assess the performance of the probes, amplicons or remanent plasma RNA from 61 deidentified clinical specimens of diverse HIV-1 subtypes genotyped by either Sanger consensus or PacBio next generation sequencing were selected, including 29 with one or more of the mutations at the four codons selected for the OLA DTG resistance probe sets (G118R, Q148H/R/K, N155H and R263K). The deidentified remanent plasma viral RNA (*n* = 22) or first-round amplicons (*n* = 39) from past Sanger or PacBio sequencing were reamplified for OLA testing. Amplification of 10 uL plasma RNA used a PrimeScript One-step RT-PCR kit (Takara Bio USA, San Jose, CA, USA) and first round primers Forward: 5′CACAGTAATTGGAGAGCAATGGCTAGTGATTTTAA and Reverse: 5′ACTTTTCCATGTTYTAATCYTCATCCTGTC, followed by a second-round nested PCR with primers Forward: 5′TAGCAAAAGAAATAGTAGCYAGCTGTGATAAATG and Reverse: 5′CAATCAKCACCTGCCATCTGTTTTCCAT (GenBank accession # pending). Leftover first-round amplicons (2 μL) from PacBio sequencing were submitted to a second-round PCR with primers Forward: 5′TCGTCGGCAGCGTCCAAATCACTCTTTGGCARCGACC and Reverse: 5′GTCTCGTGGGCTCGGCCGCTCCGTCCGACGACTCACTATA (Boyce C.L., unpublished; GenBank accession # pending). All second-round amplicons were visualized by agarose gel electrophoresis, diluted 1:4 in water and 2 μL was used for OLA testing. OLA results were compared to prior PacBio (*n* = 15) or Sanger sequencing (*n* = 46) genotypes.

## 3. Results

### 3.1. Selection of DTG-Resistance Mutations for Probe Design

Publicly available genotypic data from 76 INSTI-naïve participants who experienced virologic failure and DTG-resistance in clinical trials and case reports [7,8,11,12,18,19,29,30,31,32,33,34,35,36,37,38,39,40,41,42,43,44,45,46,47,48,49,50,51,52] were analyzed to identify the most prevalent integrase mutations and to select the fewest mutant codons that would identify the greatest proportion of individuals with DTG DR. We observed that the major INSTI mutations listed in the Stanford HIV Drug Resistance Database [26,27], G118R, Q148H/R/K, N155H or R263K, were found in 73 of the 76 (96%, 95% CI 88.6–99.1%) individuals reported to have DTG resistance at virologic failure, and occurred as single mutations in 42 (55.3%) (Table 1). The remaining three individuals had a single T66I/K, a single S147G, and a combination of T66A + E138K + Y143R + 147G, respectively. E138K/A/T was detected at a frequency of 31.6%; however, it always occurred in combination with other INSTI-resistance mutations, as did E92Q, G140S/A/C and Y143R/C/H. Based on these findings we selected mutations G118R, Q148H/R/K, N155H and R263K as the OLA DTG resistance mutations for probe design (sequences listed in Table 2, section A) and eventual inclusion in the INSTI resistance OLA kits.

### 3.2. Performance of OLA Probes to Detect DTG Resistance Mutations

The probes were evaluated using remanent RNA or amplicons from 61 clinical samples with HIV-1 subtypes A (*n* = 17), B (*n* = 16), C (*n* = 10), D (*n* = 9) and AE (*n* = 9) with prior genotypes defined by Sanger or PacBio sequencing. Among 244 codons analyzed for mutations G118R, Q148H/R/K, N155H and R263K, the OLA detected 27 mutant codons, 201 wildtype codons, and 16 (6.5%) codons with an indeterminate genotype due to failure of ligase to join the “genotype-specific” and “common” probes (Table 3). Compared to prior sequencing results, OLA detected 27/29 DTG DR mutations (93.1%). The two missed mutations were at codon 118; one had an indeterminate result by OLA and the other had a frequency of 2.8% by PacBio but tested wildtype by OLA. The OLA detected all other minority variants (<20% frequency in the viral population) quantified at 3.6%, 3.9%, 7.1%, 11.1%, 16.7% and 17.6% by PacBio. There were no wildtype genotypes misclassified as mutant by OLA (false positives) at any of the codons tested.

**Figure 1:**
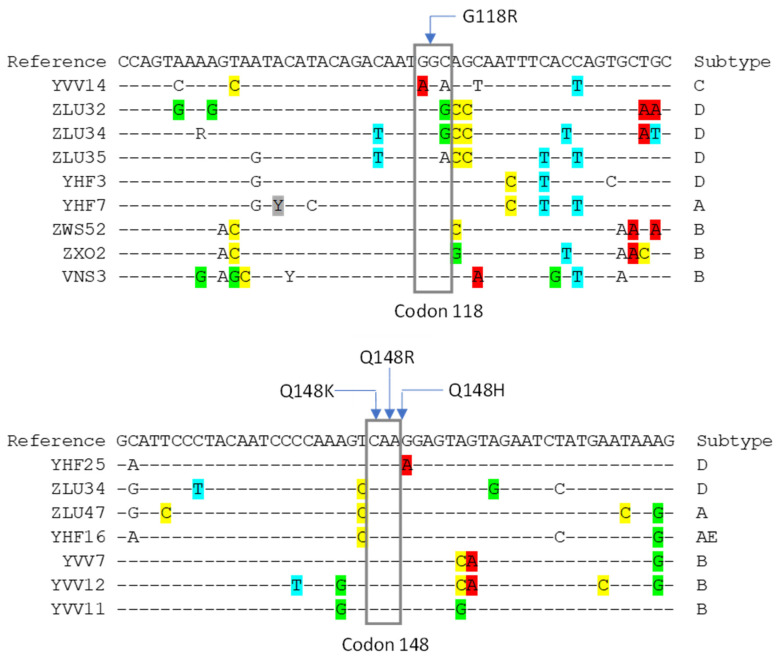
Indeterminate OLA results at HIV-1 integrase codons 118 and 148. The reference sequence shows the consensus sequence in the region of the OLA probes of all samples tested. The gray rectangles indicate the bases comprising the codon of interest; the blue arrows indicate the ligation site for each mutation tested; and bases highlighted in color indicate polymorphisms in samples with indeterminate results not accommodated by the original OLA probes.

Due to the 16 indeterminate OLA results, 9 at G118R and 7 at Q148H/K/R/ (6 at Q148R, 3 at Q148K and 2 at Q148H), the integrase sequences from samples with ligation failure at codons 118 and 148 were reviewed. An alignment of the region complementary to the OLA probes from samples with indeterminate results at codon 118 showed several polymorphisms causing mismatches with the OLA probes that likely interfered with the ligation reaction due to their proximity to the ligation site (Figure 1, top alignment): A variant coding for the arginine (R) mutation but having an “A” instead of “C” at the ligation site (sequence YVV14) and an AG→CC substitution at the 4th and 5th position from the ligation site on the common probe (sequences ZLU32, ZLU34 and ZLU35). A third substitution, a C→T at the 11th position from the ligation site on the common probe, was present in close proximity to additional polymorphisms in sequences ZLU35, YHF3 and YHF7. The alignment of sequences with indeterminate results at codon 148 (Figure 1, lower alignment) found a G→A substitution within one or two bases from the ligation site on the common probe for variants Q148H and Q148R, respectively (sequence YHF25); a T→C substitution three bases from the ligation site on the genotype-specific probe for variant Q148R (sequences ZLU34, ZLU47 and YHF16); and an AG→CA substitution in the common probe region (sequences YVV7 and YVV12; these two samples corresponded to sequential time points from the same individual). The remaining sequences with indeterminate results at codons 118 or 148 had substitutions at multiple sites that could have impaired annealing to the genotype-specific or common probes.

To address polymorphisms observed in sequences with indeterminate OLA results at codons 118 and 148, three additional probes were designed for codon 118 with modifications at the sites described above (a mutant probe for the alternate arginine variant and two common probes) and two new genotype-specific probes were made for mutation Q148R modified with a C/T mix at the third position from the ligation site (Table 2, section B). Other potentially interfering polymorphisms identified near codons 118 and 148 occurred in single samples within our panel and were uncommon in multiple sequence alignments from the Los Alamos Database; therefore, we did not design additional modified probes to address these polymorphisms.

When samples with indeterminate results at codon G118R (YVV14, ZLU32, ZLU34, ZLU35, YHF3 and YHF7) were re-tested using the modified OLA probes combined with the original set of probes, the G118R mutant with the alternate base (YVV14) had a strong mutant result, and the total number of indeterminate codons were reduced from 9 to 5 at G118R. Similarly, samples ZLU34, ZLU47 and YHF16 had wildtype results when re-tested for Q148R with the modified genotype-specific probes, reducing the number of indeterminate results from 6 to 3 at Q148R. Including the results obtained with the modified OLA probes, the overall rate of indeterminates across codons was 3.7% (9/244 codons; 5 at G118R and 4 at Q148H/K/R), the OLA sensitivity for mutant detection was 96.6% (28/29 mutants, 95%CI 82.2–99.9%) and the specificity was 100% (95%CI 98.2–100%).

## 4. Discussion

To address the emerging problem of drug resistance to DTG in RLS [13,14,15,53] compared to past clinical trials [54], we developed reagents to detect DTG DR using an economical OLA-Simple test. First, we found that mutations at four codons in the integrase gene identified 96% of individuals with DTG DR at virologic failure during DTG-ART regimens. Second, we developed and optimized OLA probes for these four codons to achieve highly sensitive and specific detection of mutants compared to sequencing, including across HIV-1 subtypes prevalent in countries with high burden of HIV infection. If further testing validates the utility of our novel OLA DTG resistance probes in additional populations, these probes could be incorporated into the OLA-Simple kit. This would allow rapid screening for DTG DR in RLS by minimally trained technicians in relatively unsophisticated laboratories, with each assay taking approximately 4 h and with supplies costing <US$20/specimen [22].

To maintain an affordable drug resistance test using a point mutation assay, the relevant mutations must be limited in number otherwise the cost and complexity of the assay escalate. We selected mutations critical to diagnosing DTG-resistance in most people by examining published genotypic data from trials and case reports of DTG DR in PLWH [7,8,11,12,18,19,29,30,31,32,33,34,35,36,37,38,39,40,41,42,43,44,45,46,47,48,49,50,51,52]. Mutations G118R, Q148H/R/K, N155H and R263 were among the most prevalent major INSTI mutations selected and occurred as a single mutation in most (55%) individuals with virologic failure of a DTG-based regimen. Thus, detection of one or more of the selected mutations captured 96% of the individuals reported to have DTG resistance among the 76 cases analyzed. A recent review that reported treatment emergent DTG mutations in INSTI-naïve individuals similarly report finding four largely non-overlapping mutational pathways characterized by integrase mutations G118R, Q148H/R/K, N155H and R263K [55]. In this report, 91 of the 99 (92%) cases who developed major DTG DR had one or more of these signature mutations, most commonly R263K or G118R. These mutations were also detected alone or in combination with other INSTI mutations in 24/26 (92%) individuals participating in the DTG RESIST study of people viremic on DTG-ART and were reported to have low-, intermediate-, or high-level resistance [10]. Together, these data support our selection of mutations to include in the OLA-Simple kit and suggest that screening for these four key mutations could identify >90% of PLWH failing DTG-ART with selection of DR who might need a regimen adjustment to suppress viral replication.

Our novel DTG resistance OLA probes detected all but one mutation quantified by PacBio sequencing at frequencies greater than 3%, a detection sensitivity similar to that observed with NNRTI OLA probes [28]. The limit of detection by the OLA was established at 2% mutant using plasmid mixtures; however, detection of mutant concentrations near the 2% cut-off appears less reliable in clinical specimens likely due to variations in PCR amplification, ligation, and/or reduced input of HIV templates into the PCR (specimens with low HIV viral load or small sample volumes) [28]. In our evaluation of the DTG OLA probes, the single mutation not detected by OLA was quantified by PacBio at 2.8% frequency in a specimen with only 72 HIV templates sequenced. Therefore, we anticipate the lateral flow format of DTG OLA will have a mutant detection sensitivity comparable to the NNRTI OLA-Simple, which had an analytical detection limit of 15% mutant and a sensitivity of 98% when compared to Sanger sequencing in clinical specimens from diverse geographic regions [22].

Our DTG OLA probes had 100% specificity for mutant detection, in agreement with our previous studies using PI, NNRTI and NRTI probes [22,28,56]. The high specificity of the OLA to discriminate between the mutant and the wildtype virus sequence is conferred by the DNA ligase which requires that the two bases flanking each side of the probes’ ligation site be perfectly complementary to the target DNA for ligation to occur [57]. In addition to its high specificity, the OLA is designed to tolerate polymorphisms in the target amplicons within the region complementary to the OLA probes by using a non-stringent temperature (37 °C) to anneal and ligate the probes. However, ligation failure can occur when mismatches between the target and probe sequences are located within 2 or 3 bases from the ligation site, and occasionally when the target DNA has multiple mismatches in the region complementary to one of the probes [56,57,58,59]. The DTG OLA probes were designed with mixed bases at several sites of common genetic polymorphisms to accommodate HIV-1 sequence diversity and thus minimize the potential for ligation failures that lead to indeterminate OLA results. The probes designed for mutations N155H and R263K performed well on our panel of clinical samples that included HIV subtypes A, B, C, D and AE, but indeterminate results were observed in several samples at codons 118 and 148. 

The HIV region complementary to the probes for detection of G118R is highly polymorphic; therefore, this set of probes was designed as mixtures of probes optimized for specific HIV subtypes to accommodate sequence variation at multiple sites. Nonetheless, several polymorphisms not addressed in the original probes appeared to cause indeterminate OLA results. Interestingly, one sample had an arginine mutant variant coded by **A**GC instead of the common **C**GC codon, which caused ligation failure due to the mismatch at the ligation site. This mutant variant was observed in a single subtype C sequence and its prevalence among mutant viruses harboring G118R is not known. Another source of ligation failure was an AG→CC substitution near the ligation site on the common probe; this double substitution occurred in 3/9 specimens with indeterminate OLA at G118R (all three were subtype D) and in the LANL Database has a prevalence of 7–15% among subtype A, B, C and G sequences. To detect the three distinct mutant variants at codon 148 (lysine (K), arginine (R) and histidine (H)), the OLA uses overlapping probes with ligation sites at different codon positions and therefore requires testing in separate OLA assays. As a result, sequence polymorphisms can affect each assay differently depending on the proximity of the mismatch to the ligation site. Q148R had the greatest proportion of indeterminate OLA results due to a relatively common T→C substitution three bases from the ligation site, which did not affect Q148H (four bases from the ligation site) or Q148K since the probe for this mutation already included a C/T mixture at this position (two bases from the ligation site). Modified probes to address these interfering polymorphisms at or near codons G118R and Q148R reduced the frequency of indeterminate OLA results at these codons. However, given that these regions of HIV-1 are highly polymorphic, testing of additional clinical specimens may find that further sequence optimization is needed. Degenerate (dK, dP) [60,61,62] and/or universal bases (dI, 5-nitroindole) [63,64] have been used in place of mixed bases to accommodate sequence variation without significantly increasing degeneracy of primers or probes. We are currently exploring this approach to design optimized probes for G118R.

This study had multiple limitations. Foremost is the small number of mutant clinical samples available to validate the DTG probes within and across subtypes. As more specimens from individuals failing DTG regimens become available, further testing will be needed to establish the detection sensitivity at each mutant codon and identify potential alternate mutant variants that may require new or modified probes prior to integration into the OLA-Simple kit. Secondly, it is not known whether specific mutations or polymorphisms among HIV-1 subtypes may influence the development of different resistance pathways. A recent study in Ethiopia found similar genetic barriers to resistance at major INSTI resistance positions in subtypes C and B, except for a lower selection of the Q148H/R/K pathway in subtype C [65]. Conversely, past in-vitro studies observed selection of G118R in subtypes CRF02 AG and C but not in subtype B viruses [66]. However, our analysis of INSTI mutations in PLWH with failure of DTG-ART included cases representing diverse HIV subtypes and geographical regions that showed selection of G118R in individuals infected with subtypes B [7,10], CRF01_AE [52], F [18] and C [7,11,12]. Third, in vitro studies have reported mutations in and near the 3′ polypurine tract (3′PPT) [67] and mutations in the envelope glycoprotein [68,69] to confer INSTI resistance; however, their frequency in persons with virologic failure while on DTG treatment has not been determined. The current OLA amplifies regions of HIV *pol*; therefore, testing for mutations outside of integrase by OLA-Simple would require amplification of additional regions of the HIV genome. If emergence of non-integrase mutations conferring DTG resistance is confirmed in persons with treatment failure, a multiplex amplification step could be added to the OLA-Simple workflow. 

In summary, we identified four codons in the HIV integrase gene that will detect nearly all individuals who develop DTG DR while receiving DTG-ART and we developed and validated a set of OLA DTG resistance probes to detect these critical DTG DR mutations. In response to the global shift to DTG-based regimens, inclusion of these OLA DTG resistance probes in the OLA-Simple kit will expand its utility for drug resistance testing in RLS. Recent reports of INSTI resistance selected in individuals failing DTG-ART suggest that increased access to affordable and timely DR monitoring for individual care is needed to minimize the emergence of INSTI resistance and thus preserve the long-term efficacy of DTG-based regimens.

## Figures and Tables

**Table 1:** Integrase mutations detected in individuals with virologic failure of DTG-based ART (*n* = 76).

Detection Rate	Major Integrase Inhibitor (INSTI) Resistance Mutations (Stanford HIV Drug Resistance Database ^1^)									
	T66IK	E92Q	G118R	E138KAT	G140SAC	Y143RCH	S147G	Q148HRK	N155H	R263K
Frequency (%)	13 (17.1)	2 (2.6)	26 (34.2)	24 (31.6)	5 (6.6)	1 (1.3)	6 (7.9)	11 (14.5)	8 (10.5)	37 (48.7)
Number (%) detected as single mutations	1 (1.3)	0	7 (9.2)	0	0	0	1 (1.3)	3 (3.9)	3 (3.9)	29 (38.2)

^1^ Mutations in bold reduce susceptibility or virological response to DTG; mutations in plain text contribute to reduced susceptibility in combination with other INSTI-resistance mutations [26,27].

**Table 2:** OLA probes designed to detect mutations in HIV-1 integrase associated with resistance ^a^ to dolutegravir.

Codon	Mutation	Probe Type	Sequence (5′ →3′) ^b^
A. Original probes			
118	G118R	Subtype A/AE wildtype	dig-CCAGTAAAARTARTACACACAGACAAY**G**
		Subtype C/D wildtype	dig-CCAGTMAAARTARTACATACAGACAAT**G**
		Subtype A/AE mutant	f-CCAGTAAAARTARTACACACAGACAAY**C**
		Subtype C/D mutant	f-CCAGTMAAARTARTACATACAGACAAT**C**
		Common	p-**GH**AGCAATTTCACCAGYRCTGC-bio
		Subtype C common	p-**GH**AGTAATTTCACCAGTRCTGC-bio
148	Q148K	Wildtype	dig-GRATTCCCTACAATCCCCAAAGY**C**
		Mutant	f-GRATTCCCTACAATCCCCAAAGY**A**
		Common	p-**AR**GGAGTAGTAGAATCYATGAATAA-bio
	Q148R	Wildtype	dig-ATTCCCTACAATCCCCAAAGT**CA**
		Mutant	f-ATTCCCTACAATCCCCAAAGT**CG**
		Common	p-**R**GGAGTAGTAGAATCYATGAATAAAG-bio
	Q148H	Wildtype	dig-TTCCCTACAATCCCCAAAGT**CAR**
		Mutant	f-TTCCCTACAATCCCCAAAGT**CAC**
		Common	p-GGAGTAGTAGAATCYATGAATAAAG-bio
155	N155H	Wildtype	dig-GTCAAGGAGTAGTAGAATCYATR**A**
		Mutant	f-GTCAAGGAGTAGTAGAATCYATR**C**
		Common	p-**AY**AAAGAATTAAAGAAAATYATAGGRC-bio
263	R263K	Wildtype	dig-TGACATAAARGTAGTRCCAAGR**AG**
		Mutant	f-TGACATAAARGTAGTRCCAAGR**AA**
		Common	p-**R**AAAGCAAARATCATTAGGGATTAT-bio
		Subtype C common	p-**R**AAAGYAAAAATCATTAAGGACTATG-bio
B. Modified probes ^c^			
118	G118R	Alt mutant	f-CCAGTAAAAGTARTACAYACAGACAAY**A**
		Mod common	p-**GH**AGCAATTTYACCAGYGCTGC-bio
		Alt common	p-**GN**CCYAATTTCACCAGTRCTGC-bio
148	Q148R	Mod wildtype	dig-ATTCCCTACAATCCCCAAAGY**CA**
		Mod mutant	f-ATTCCCTACAATCCCCAAAGY**CG**

^a^ References [7,8,11,12,18,19,26,27,28,29,30,31,32,33,34,35,36,37,38,39,40,41,42,43,44,45,46,47,48,49]. ^b^ Bases comprising the codons of interest within the genotype-specific and the common probes are in boldface type; dig, digoxigenin; f, fluorescein; p, phosphate; bio, biotin. ^c^ Probes with modifications designed to accommodate polymorphisms detected in samples with indeterminate results in OLA.

**Table 3:** Summary of genotypes derived by OLA versus PacBio/Sanger sequencing at 244 HIV-1 integrase resistance codons.

Mutation	OLA *n* = 244			PacBio/Sanger *n* = 244	
	Mutant	Wildtype	Indeterminate	Mutant	Wildtype
G118R	6	46	9	8	53
Q148K/R/H *	7	47	7	7	54
N155H	7	54	0	7	54
R263K	7	54	0	7	54
Total	27	201	16	29	215

* Because the position of the mutant base differs in Q148K, Q148R and Q148H, each variant was tested in a separate OLA assay. Interpretation of codon 148 for each specimen was as follows: mutant-positive if any of the three variants were detected; wildtype if all three assays produced wildtype results; and indeterminate if no mutant variant was detected and at least one variant was negative for mutant and wildtype. All seven mutations detected by PacBio/Sanger at codon 148 were detected by OLA and included three Q148K, two Q148R and two Q148H.

## Data Availability

The viral sequences analyzed in this study will be deposited in GenBank and their accession numbers will be provided. Other data presented in the study are included in the article, further inquiries can be directed to the corresponding author/s.
